# Our evolved understanding of the human health risks of mercury

**DOI:** 10.1007/s13280-023-01831-6

**Published:** 2023-02-15

**Authors:** Niladri Basu, Ashley Bastiansz, José G. Dórea, Masatake Fujimura, Milena Horvat, Emelyn Shroff, Pál Weihe, Irina Zastenskaya

**Affiliations:** 1 McGill University, 204 CINE Building, Ste. Anne de Bellevue, Montreal, QC H9X 3V9 Canada; 2 Faculdade de Ciencias da Saude, Universidade de Brasília, Brasília, 70919-970 Brazil; 3 National Institute for Minamata Disease, Minamata, Kumamoto 867-0008 Japan; 4 Department of Environmental Sciences, Jožef Stefan Institute, Ljubljana, Slovenia; 5 Public Health Authority of Seychelles, Mont Fleuri, Mahe, Seychelles; 6 Department of Occupational Medicine and Public Health, Sigmundargøta 5, 100 Tórshavn, Faroe Islands; 7 WHO European Centre for Environment and Health, Platz Der Vereinten Nationen 1, 53113 Bonn, Germany

**Keywords:** Environmental sciences, Epidemiology, Methylmercury, Public health, Review, Toxicology

## Abstract

Mercury (Hg) is a chemical of health concern worldwide that is now being acted upon through the Minamata Convention. Operationalizing the Convention and tracking its effectiveness requires empathy of the diversity and variation of mercury exposure and risk in populations worldwide. As part of the health plenary for the 15th International Conference on Mercury as a Global Pollutant (ICMGP), this review paper details how scientific understandings have evolved over time, from tragic poisoning events in the mid-twentieth century to important epidemiological studies in the late-twentieth century in the Seychelles and Faroe Islands, the Arctic and Amazon. Entering the twenty-first century, studies on diverse source-exposure scenarios (e.g., ASGM, amalgams, contaminated sites, cosmetics, electronic waste) from across global regions have expanded understandings and exemplified the need to consider socio-environmental variables and local contexts when conducting health studies. We conclude with perspectives on next steps for mercury health research in the post-Minamata Convention era.

## Introduction

Mercury is a chemical pollutant of human health concern worldwide. The World Health Organization has listed mercury as one of its top 10 chemicals of public health concern (WHO 2020). International assessment projects have documented that: (1) sources of mercury pollution are globally distributed; (2) emission levels continue to increase over time; and (3) these emissions are largely due to anthropogenic activities (UNEP 2019a). The levels of mercury in certain aquatic and food items, consumer and industrial products, and occupational settings can be at levels deemed concerning to human health (UNEP & WHO 2008). Human biomonitoring studies have established that most people worldwide are exposed to some amount of mercury, and that there are notable vulnerable groups (e.g., Indigenous Peoples, artisanal and small-scale gold miners (ASGM)) due to dietary or occupational factors (Basu et al. 2018).

Mercury and its compounds have many properties that make them useful in society, though balancing these essential uses against the metal’s toxic properties proves challenging. In Goldwater’s historical examination, diverse uses of mercury and its compounds were tracked back several hundred years BC in China, India, and across the Arabic world (Goldwater 1972). Alongside these, early uses were many recorded instances of human poisoning events, largely within the artisanal occupational sector (e.g., alchemists, felt hat producers) or within medical practices (e.g., treatment of syphilis and skin infections). For example, Scopoli (1771) precisely described the symptoms and signs of occupational poisoning with elemental mercury in mining sites and specifically mentioned the “unusually sad mental state of these workers.” As noted by Woodall in 1639, mercurous or quicksilver is: “The hottest, the coldest, a true healer, a wicked murderer, a precious medicine, and a deadly poison, a friend that can flatter and lye” (Woodall, n.d).

In the mid-twentieth century, catastrophic events at Minamata Bay and elsewhere signaled to the world that mercury pollution remains of great contemporaneous concern. Since then, notable epidemiological studies from the Faroe Islands, the Seychelles, New Zealand, the Amazon, and the Arctic have demonstrated that mercury is of neurodevelopmental concern with growing evidence of impacts on the cardiovascular and immune systems. Collectively, there is a tremendous body of knowledge concerning the impacts of mercury on human health, and this has been summarized in recent exemplary review papers (Ha et al. 2016; Basu et al. 2018; Eagles-Smith et al. 2018).

The Minamata Convention on Mercury entered into legal force on August 16, 2017, and committed to control the supply and trade of mercury (UNEP 2019b). This multilateral agreement exemplified a global commitment to protect human health and the environment from anthropogenic sources of mercury. Article 16 of the Convention encourages Parties to promote strategies to: (1) identify and protect populations at risk; (2) adopt health guidelines regulating mercury exposure; (3) develop and implement educational and preventive programs; and (4) establish and strengthen capacities to prevent, diagnose, treat, and monitor health risks (UNEP 2019b). The expectation of those who crafted the Convention is that by limiting the use and environmental release of mercury, while also promoting activities such as education, capacity building, and knowledge sharing, will improve the health of humans and the environment over time. However, the reality will undoubtedly be more complicated and nuanced. Operationalizing the Convention and tracking its effectiveness will require empathy and understanding of the diversity and variation of mercury exposure, risk scenarios, and socio-environmental aspects in populations worldwide. Mercury is often found in association with phenomena that have high societal and economic value (e.g., food safety and security, mining and economic development, dentistry, and oral health), but these situations vary worldwide.

Our understanding of mercury’s risk to human health continues to evolve (Table 1). There was an early focus on workplace safety and environmental disasters led by scholars of occupational health, medicine, and toxicology, and this was followed by studies of select vulnerable population groups led by epidemiologists and exposure scientists. Collectively, studies in these aforementioned areas have formed an extensive knowledge base from which robust evidence was leveraged to justify the Minamata Convention. Moving forward, we continue to realize that there are complex social and environmental contexts within which mercury is found, and we posit that these are best understood through multidisciplinary (e.g., medicine, toxicology, epidemiology), inter-sectoral (e.g., academia, industry, government), and participatory (e.g., community members and advocates) approaches that in particular involve those from the social sciences and global public health communities. Accordingly, the objective of this review paper is to help mercury scientists and other stakeholders (e.g., those involved with the Minamata Convention, NGOs, and communities vulnerable to mercury pollution) to gain a deeper and more holistic understanding of the health risks associated with mercury. In particular, this paper reviews how our scientific understandings have evolved over time and showcases contemporaneous source-exposure scenarios from across global regions that exemplify the need to consider socio-environmental variables and diversity when conducting health studies.

**Table 1:** Evolution in our understanding of mercury (Hg) and health organized on a quarter-century basis

	1950–1975	1975–2000	2000–2025	2025–onwards
Driving discipline	Occupational Medicine	Toxicology	Epidemiology	Systems Science
Focused population	Acute poisoning events	Cohort studies on highly exposed groups	Cross-sectional and other studies on groups exposed to diverse sources (e.g., ASGM, Hg containing products)	All population groups
Population size of study groups	Very small (10 s to 100 s);	Small (100 s to 1,000 s)	Moderate (1,000 s to 10,000 s)	Large (10,000 and up)
Geographic settings	Households or workplaces	Community-level (few studies)	Community-level (many studies, including expansion into more vulnerable settings), with regional considerations	National and regional (especially low- and middle-income), with global considerations
Geographic regions	Scattered	Few, largely in high-income regions	Expansion across Asia Pacific, Africa, Latin America	Global focus, while keeping attention on local to regional
Exposure considerations	Acute, high dose exposures to Hg	Chronic, lower dose exposures focused on MeHg derived from seafood consumption	Focus on different sources of exposure (e.g., ASGM, other food items) and chemical forms of Hg	Integrative understandings, that start to consider co-stressors, social factors, Hg-Hg interactions, etc
Exposure assessment	Interview based	Dietary (and other) surveys to gauge intake, human biomonitoring	Human biomonitoring	Exposure modeling
Health study types	Case reports, few cross-sectional studies	Birth cohort studies, more cross-sectional studies	Complex epidemiological studies to reveal mercury effects	Public health studies that prioritize prevention and interventions
Health outcomes	Adverse clinical symptoms	Sub-clinical effects (neurodevelopment)	Molecular to sub-clinical effects (neurodevelopment, and other physiological systems including cardiovascular and immune)	Integrative understandings, that start to consider co-stressors, social factors, life course aspects, etc

## Method

The authors of this paper were invited by the organizing committee of the 15th International Conference on Mercury as a Global Pollutant (July 2022, virtual format) to serve on the Plenary Panel on Human Health. The co-authors represent a diverse team of experts (disciplines, research interests, career stages, geographic regions). Through late 2021 and into 2022, discussions were facilitated through online meetings and email exchanges. Key events from which feedback on this paper was obtained from stakeholders included the following: (a) a “Minamata Online” presentation to the global community on October 19, 2021^1^; (b) the plenary presentation at the 15th International Conference on Mercury as a Global Pollutant on July 26, 2022; and (c) participation in the International Conference on Mercury as a Global Pollutant synthesis workshop on September 1 and 2, 2022.

This paper is classified as a “critical review” based on the typology outlined by Grant and Booth (2009) as our work aims to identify the most important events in the field in a narrative and conceptual model. The work is presented temporally along a quarter-century basis to help organize our evolved understandings (Table 1). The first and second parts of the paper briefly reflect on important case studies through the mid- and late-twentieth century that range from acute poisoning events to focused cohort studies from select locations. The third part of the paper presents upon diverse source-exposure scenarios nowadays from across global regions that exemplify the need to consider socio-environmental variables. The final part of the paper concludes with proposed next steps to conduct mercury health research in the post-Minamata Convention era.

## 1950 to 1975: An era of tragic mercury poisoning events

The postwar period at the end of the 1940s coincided with a defining industrial event involving mercury pollution—the Minamata Bay disaster. This event was among the first and most serious contamination events that helped define the modern environmental awareness movement. Minamata disease was first officially recognized in the Japanese city of Minamata (Kumamoto Prefecture) in 1956 (Ekino et al. 2007). The Minamata factory, owned by the Chisso Corporation, was a major industrial plant in Japan that produced various chemical products. One such chemical was acetaldehyde, which generates methylmercury as a by-product. The factory’s effluent contained methylmercury which ended up contaminating Minamata Bay. Many consumers of fish and shellfish from Minamata Bay developed Minamata disease. A second Minamata disease (called Niigata Minamata disease) was later reported in 1965 in Niigata Prefecture where the Showa Denko Kanose Plant released methylmercury into the Agano Basin.

Some of the main symptoms of Minamata disease include loss of sensation in hands and feet, narrow vision, hearing difficulty, and slurred or unclear speech. The severity of symptoms varies depending on the individual. In the initial period of the outbreak, among individuals with severe symptoms, some experienced convulsions, loss of consciousness, and death (Takeuchi 1982). Some babies were born with the symptoms of Minamata disease (“fetal Minamata disease”). Neurodevelopment outcomes in children that were born from mothers consuming mercury-contaminated fish have been well documented (Harada et al. 1999). These observations raised concerns over the safety of fish consumption (especially during pregnancy), and spurred investigations in other parts of the world as outlined in the next section.

In addition to the events in Minamata, another infamous mass poisoning took place in Iraq during the winter of 1971–1972. Seed grain treated with a methylmercury fungicide was used to prepare homemade bread in rural communities throughout Iraq (Bakir et al. 1973). Total hospital admissions increased to over 6000, with most individuals being admitted in January 1972. Around 460 deaths attributed to methylmercury were recorded in hospital (Bakir et al. 1973). Aside from these well-known environmental disasters, there were many reports of mercury poisoning events from across the world. For example, Cappelletti et al., (2019) identified 45 published papers (consisting of 174 cases) across the medical literature worldwide over the time frame of 100 years where exposure to mercuric chloride was attributed to intoxication or death.

## 1975 to 2000: Toxicological studies on highly exposed populations

The aforementioned environmental disasters and poisoning events raised concerns about the presence of methylmercury in fish and seafood, and as such starting around the 1980s epidemiological research became quite active in several areas, especially in the Seychelles and Faroe Islands, as well as in the Arctic and Amazon. Collectively these studies established associations between methylmercury exposures, particularly in early life, and adverse health outcomes with an initial focus on neurodevelopment (Table 1). Additionally, these studies started to demonstrate that a range of socio-environmental factors in addition to mercury exposure are important when conducting health studies (Table 2).

**Table 2:** Characteristics of major mercury health studies conducted in the latter stages of the twentieth century

Location	Main source of mercury exposure	Amount of relevant food consumed	Blood total mercury levels ^a^	Key birth cohort studies	Key socio-environmental factors
Faroe Islands	Ocean fish and marine mammals	Pilot whale meat is the main mercury source; consumption can vary from none (women of child-bearing age) to ~ 15 kg/yr in elderly men	1987 (*n* = 1,022): 22.3 ug/L 1995 (*n* = 182): 21.0 ug/L 1999 (*n* = 475): 12.4 ug/L 2008 (*n* = 500): 4.6 ug/L	The Birth Cohort Studies of the Faroe Islands	Whaling has been a part of Faroese culture for centuries and an important contribution to the nutrition in the past
Seychelles	Ocean fish	57 kg of ocean fish per person per year	1989 (*n* = 779): 23.6 ug/L 2001 (*n* = 300): 22.5 ug/L 2008 (*n* = 1,265): 11.7 ug/L	Seychelles child development study	Nutritional benefits associated with consumption of ocean fish
Arctic	Marine mammals and fish	Ringed seal liver (2 kg) and muscle (8 kg), and Arctic char (20 kg) per person per year	8.6 μg/L (IQR:3.6–16.2 μg/L) *n* = 7,472	Various studies	Dietary transition driven by economic considerations, climate variability, and public health messaging
Amazon	Riverine fish	11 to 148 kg/yr per person	15.4 μg/L (IQR: 8.7–32.9 μg/L) *n* = 18,509	Various studies	Diverse mercury exposures that bring together occupational and environmental considerations

^a^Biomonitoring data extracted from UN Global Mercury Assessment 2018 (Basu et al. 2018)

### Seychelles

The Seychelles child development study was designed to investigate the adverse effects of methylmercury exposure on children’s neurodevelopment. The Seychelles is a small-island state, and its residents consume about 57 kg of fish per person per year. These fish contain varying levels of methylmercury, but most fish commonly consumed have concentrations below 0.5 μg/g, a level comparable to commercially available fish in developing countries. Fish is not only an important source of protein in their diet but also forms part of their cultural and economic activities. The high fish consumption in this nation makes it an ideal place to conduct a large-scale population study to assess the effect of mercury exposure from fish consumption. The Seychelles is advantageous as an epidemiological site since there are minimal industries that cause environmental pollution, consumption of sea mammals (i.e., animals with highest level of methylmercury bioaccumulation) is illegal, and education and healthcare are universally free and accessible (Govinden et al. 2020).

Mothers in the main cohort (779 mother infant pairs recruited in 1989–1990) had hair methylmercury concentrations in the range of 1–40 μg/g with a mean of 6.8 μg/g. There were no adverse associations found between methylmercury measured in maternal hair during pregnancy and child neurodevelopment up to 66 months of age. The findings that children with higher prenatal exposure showed better scores than children exposed to lower methylmercury levels on some tests suggested that some unmeasured variables such as nutrition might be playing an important role (Myers et al. 2000). Further evaluation of children at 107 months found no clear pattern of adverse association with prenatal methylmercury exposure (Myers et al. 2000).

There have been two additional cohorts with over 1600 mother–child pairs recruited, and further studies are being conducted to understand possible health effects at prenatal or postnatal stages as a consequence of methylmercury exposure through consuming a high fish diet (Strain et al. 2015; Davidson et al. 2008). It should be noted that the children in the different cohorts undergo extensive examination for neurodevelopmental assessments. Test batteries assess multiple neurodevelopmental domains including cognition, psychomotor development, social communications, language development and executive functions. Additionally, analysis of school achievements, including a standardized academic assessment among Southern African countries, showed a high achievement by the Seychelles cohorts and no adverse mercury effects (Leste and Davidson 2020).

Studies over the last 30 years have shown no adverse effect on neurodevelopmental outcomes among Seychellois children and young adults up to the age of 19 years, from their mother’s or their own consumption of fish. The absence of adverse effects over several decades and cohorts, suggests that other factors could be involved in providing a protective effect or enhancing development, and that the levels of mercury in the fish consumed are too low to have harmful effects on behavior and development. Consequently, some of the cohorts recruited have explored the nutritional benefits of eating fish. Results highlighted that n3 and n6 Poly Unsaturated Fatty Acids (PUFAs) may be playing a key role in modifying the influence of methylmercury, especially the function of n3 fatty acids being important for brain development (Strain et al. 2008, 2012). The role played by selenium in sequestering methylmercury may also help in reducing the potential toxic effects (Nuttall 1987).

Additionally, over 70 species of fish relevant to the Seychelles’ diet have been analyzed for essential nutrients (e.g., trace elements, fatty acids, proteins) and for contaminants, not only focusing on mercury but also on cadmium, lead, and persistent organic pollutants (POPs) (Sabino et al. 2022). Fish located in diverse marine areas are rich in essential trace elements, amino acids and omega-3 fatty acids which have a variety of health benefits. In terms of POPs, all were found to be lower than the EU limit and much lower than in fish found in the Mediterranean and Atlantic Ocean. Heavy metals in fish tested were all below the EU limits and at levels that do not present any risk for human health.

### Faroe Islands

In 1986, a birth cohort study of 1022 participants was established in the Faroe Islands with the goal of gauging dietary methylmercury exposure (mainly from traditional consumption of pilot whale meat) and neurodevelopmental outcomes (Grandjean et al. 1992). Children’s prenatal exposure was assessed from the mercury concentration in cord blood, and maternal hair mercury concentrations were also determined. Studies from this cohort determined cognitive effects at age 7 years could be associated with prenatal exposures (Grandjean et al. 1997), and that such effects could persist through to 14 years of age (Yorifuji et al. 2011). Clinical examinations of 847 cohort members at 22 years of age were carried out in 2008–2009 using a panel of neuropsychological tests that reflected major functional domains. Deficits in Boston Naming Test (BNT) and other tests of verbal performance were significantly associated with the cord blood mercury concentration (Debes et al. 2016). Deficits were also present in all other tests applied, although most were not of statistical significance. Structural equation models were developed to ascertain the possible differences in vulnerability of specific functional domains and the overall association with general intelligence. All models for individual domains showed negative associations, with mercury-associated changes with crystallized intelligence being highly significant. There was also a highly significant negative association between mercury exposure and general intelligence (based on all domains), with an approximate deficit that corresponds to about 2.2 IQ points at a tenfold increase in prenatal methylmercury exposure. Although the cognitive deficits observed were smaller than examinations at younger ages, maternal diets with contaminated fish and seafood were associated with adverse effects in this birth cohort at age 22 years. The deficits affected major domains of brain function as well as general intelligence. Thus, based on findings from the Faroe Islands cohorts, prenatal methylmercury exposure appears to cause permanent adverse effects on cognition (Debes et al. 2016).

Through these cohort studies at the Faroe Islands, two important dimensions became evident; whaling culture and the dietary transition away from marine food containing high levels of mercury. First, whaling has been an important contribution to the nutrition of people in the Faroe Islands, especially in times of insufficient food supply (AMAP 2021). The distribution of the meat and blubber to each household was regulated in detail. Currently, the food supply from local fishing and farming and from import markets has eliminated the nutritional need for whaling. However, whaling is still an important part of the cultural identity of the inhabitants though socioeconomically insignificant.

Second, the role of dietary transition has been notable in the Faroe Islands largely driven by increased importing ability and household purchasing power (AMAP 2021). The intake of fish has been steadily decreasing from about 4 meals to under two meals per week since the 1980s. Health authorities have offered dietary advice to educate the public about the nutritional values of fish consumption and warned against eating contaminated marine food including whale meat and blubber as well as information on fish that were low in contaminants and high in nutrients (AMAP 2021). The dietary advice from the Faroes Board of Health has been effective for pregnant women in terms of helping reduce mercury exposures, which was demonstrated in large birth cohort studies where mercury concentrations in umbilical cord blood have decreased from approximately 22.3 µg/L in 1987 to 4.6 µg/L in 2008 (AMAP 2021).

### Arctic Inuit

The effects of prenatal mercury exposure have been studied in several communities in the Arctic as reviewed in the 2021 Arctic Monitoring and Assessment Programme report on mercury (Basu et al. 2022). A study in Nunavik children at age 11 years showed that mercury exposure was associated with weaker early processing of visual information and memory functions, lower estimated IQ, poorer comprehension and perceptual reasoning, and increased risk of attention problems and ADHD behavior (Boucher et al. 2009, 2010, 2011; Boucher et al. 2012a, b; Boucher et al. 2012a, b). Conflicting results have been reported regarding the impact of prenatal mercury exposure on blood pressure with 7-year-old Faroese children exhibiting elevated blood pressure, compared to children from Nunavik, showing no association between blood pressure and prenatal mercury exposure (Grandjean et al. 1997). However, elevated blood pressure was found to be associated with mercury exposure among adults from the Faroe Islands and Nunavik. Decreased heart rate variability was associated with cord blood mercury concentrations in Faroese children at age 7 and age 14 years but not in children at age 11 years from Nunavik. Contemporary blood mercury concentrations in these children from Nunavik were associated with decreased overall heart rate variability parameters, which were also the case for adults from Nunavik and for James Bay Cree.

Research from the region documents that blood mercury levels measured in pregnant women in the Arctic have declined steeply since the 1990s, but that levels in Nunavik and Greenland remain 4 to 5 times higher than those in other Arctic regions due to change in diet. The health impacts of such dietary transitions in the Arctic are complex (Basu et al. 2022). Studies from the Faroe Islands (Basu et al. 2022) and the Inuit Health Survey of 36 communities in Arctic Canada (Rosol et al. 2016) clearly show that northern populations are consuming less traditional foods such as fish, whale, and seal meat. There are multiple factors driving this transition, with notable ones being economic hindrances, climate change influences on species distribution and biodiversity, and contaminant-related consumption advisories issued by health authorities or that spread through social media. While the reduced consumption of certain traditional foods has been associated with reductions in mercury exposure, researchers have observed concerning trends associated with a shift toward a market diet coupled with a more sedentary lifestyle. Specifically, rates of obesity, diabetes, and other metabolic diseases have been increasing in many communities. The increased reliance on store-bought foods has also raised concerns about food security in these communities.

### Amazonian region

The Amazonian region is one that represents a major source of mercury emissions worldwide, but also one in which human exposures are of potential concern. In terms of emissions, mercury is widely used in the amalgamation process for silver and gold mining. From the 1980s, Brazil has been one of the largest of producers of gold in South America, with approximately 90% coming from small-scale gold mining or “garimpos” (Malm 1998). The largest “garimpo” region is found near the Tapajós River basin in Brazil, with the peak production in 1989 reaching 4 tons of gold per month and emitting up to 120 tons of mercury annually throughout the 1990s (Veiga 1997). Despite a decline in gold production in this region, the miners and community members in this area have been exposed to mercury since at least the 1980s and living in contaminated environments (Berzas Nevado et al. 2010). High concentrations of mercury have been identified in the soil composition in the Amazon, and increased soil erosion from human activities (i.e., gold mining, forest clearing) is a source of mercury in local aquatic systems (Roulet et al. 1998, 1999).

In the review by Berzas Nevado et al. (2010), blood mercury concentrations in fish-eating populations varied from 36.1 to 90.4 µg/L. Recent studies continue to report on relatively high levels of blood mercury from these populations (i.e., range 19.6–105.0 µg/L) (Mendes et al. 2021). These blood mercury values among fish consumers are higher than those reported (12.2 µg/L) in gold-prospectors or garimpeiros (Berzas Nevado et al. 2010). Though, in the urine samples of these miners, mercury levels ranged from 4 to 450 µg/L which is more reflective of their exposures to inorganic/elemental forms of mercury. Similarly, while hair mercury (reflective of methylmercury exposure from the diet) was higher in fish-eating populations, urine mercury concentrations were higher among garimpeiros (Barbosa et al. 1997).

Frequency of fish intake in subsistence populations are for the most part reported daily, and some families may have more than one meal containing fish per day. The mean daily fish consumption as reviewed recently may range from 30 to 406 g/day for different types of populations and measured by different methods (Mendes et al. 2021). Furthermore, among traditional villagers, mercury hair concentrations in traditional mothers are good predictors of family mercury hair concentrations (Mendes et al. 2021).

In relation to the reference doses, human exposure to mercury is strongly associated to fish consumption in the Amazon region. The Amazon rain forest has a high methylating potential which makes methylmercury a prevalent component in fish-based diets. Therefore, in comparison with populations consuming commercial fish, methylmercury appears as a food component that poses special challenges in traditional high fish consumers. In these populations, it is difficult to identify a specific negative health condition directly related to fish consumption amidst a myriad of stressors such as parasitic infections and a threat of food insecurity (Dórea and Marques 2016). Additionally, Amazonian infants are exposed to methylmercury from maternal fish consumption and ethylmercury from thimerosal-containing vaccines (Marques et al. 2016). For example, during the first 6 months an exclusive breastfed infant can receive an estimated 279.2 µg of mercury in breast milk in addition to 150 µg of mercury (as ethylmercury from Thimerosal-containing vaccines) (Dórea 2007). Moreover, mercury fish advisories may unintendedly cause undue anxiety and confusion regarding real, rather than perceived, health threats to subsistence fish consumption. However, there are beneficial aspects to fish consumption, as noted above in the examples from the Seychelles and Arctic regions. Nevertheless, neurological issues related to mercury exposure through occupational routes and fish consumption remain of concern in the Amazonian region (Dórea & Marques 2016).

## 2000 to current: Contemporary investigations on diverse source-exposure scenarios

The earlier sections reviewed health studies based on acute and high occupational exposures, and environmental disasters ("1950 to 1975: An era of tragic mercury poisoning events" section). These then spurred epidemiological studies of select vulnerable population groups to establish a strong evidence concerning the adverse human health impacts of mercury pollution ("1975 to 2000: Toxicological studies on highly exposed populations" section). Taken together, these studies (largely conducted in the twentieth century) have established the following four points:

- Acute exposures to high levels of all mercury compounds can be fatal.
- Chronic exposure to methylmercury (at relatively high levels) is associated with adverse neurodevelopmental outcomes, as well as cardiovascular risks.
- Methylmercury-contaminated fish, seafood, marine mammals, and other items represent a critical dietary pathway of exposure, though many of these same food items provide health and social benefits which complicate risk assessment and management.
- There are notable population groups vulnerable to mercury either due to exposure pathways (e.g., Indigenous Peoples) or physiological factors (e.g., pregnant women and newborns).

As we entered the twenty-first century, and with continued research, it has become clearer that the health impacts of mercury extend beyond the points above (besides fish and seafood, there are other food items of concern; beyond pregnancy and fetal stages, all life stages are vulnerable to mercury, etc.) (UNEP and WHO 2008; Ha et al. 2016). There is greater awareness of the complex socio-environmental contexts within which mercury is found (Eagles-Smith et al. 2018), and we posit that these are best understood through multidisciplinary (e.g., medicine, toxicology, epidemiology), inter-sectoral (e.g., academia, industry, government), and participatory (e.g., community members and advocates) approaches that involve those from the social sciences and global public health communities.

In this section, we present key “sources” of contemporaneous mercury exposure and risk to human populations that are garnering more attention. We also discuss these from different perspectives so as to demonstrate the importance of considering diversity and variance when trying to understand mercury risk in populations worldwide and a need to focus on context-specific data.

### Food (including fish) intake

It is well established that the major pathway of methylmercury exposure to most populations worldwide is dietary and commonly through the consumption of contaminated fish and seafood. Mercury released into the environment can be converted by microorganisms to methylmercury, which bioaccumulates and biomagnifies through the food web, particularly in aquatic systems (Obrist et al. 2018). Sampling of seafood has found widespread methylmercury contamination (GEMS/Food Contaminants 2021), with some widely consumed predatory species including tuna, swordfish, grouper, mackerel being among the most highly contaminated. Therefore, for many communities, dietary consumption of contaminated fish, shellfish, and marine mammals is an important source of exposure. Unfortunately, seafood is the main source of protein for billions of people worldwide (FAO 2020). Other foods, such as rice, grown in sites heavily contaminated with mercury may also represent a source of both organic and inorganic mercury exposure for some communities to mercury (Miklavčič et al. 2013; Rothenberg et al. 2014). Finally, in some populations (e.g., Inuit), the consumption of marine mammal tissues is the major source of mercury exposure (Basu et al. 2022).

#### A need for site- and fish-specific data

Though we have known for a long time that fish consumption is a major driver of mercury exposure into most populations worldwide, many parts of the world lack data on mercury levels in fish and other food items (UNEP 2019a). The World Health Organization recognizes the importance of collecting site-specific data of mercury concentrations in fish and seafood from around the world as there is a high variation in levels across and within species. To help fill data gaps, we can examine the work of Lavoie et al. (2018) who analyzed global marine fisheries trade flows coupled with knowledge on mercury levels in 41 fish groups in the FAO’s 'International Standard Statistical Classification for Aquatic Animals and Plants' (ISSCAAP) to estimate the mean per capital methylmercury intake for each country. The highest intake value was recorded for the Maldives (23.0 μg/kg bw/week) followed by Kiribati (9.0 μg/kg bw/week), Iceland (8.0 μg/kg bw/week), Samoa (6.0 μg/kg bw/week) and Malaysia (5.9 μg/kg bw/week). On a regional basis, the highest values were recorded in countries located in the South-East Asia and Western Pacific regions, according to the WHO Classification. Overall, the researchers concluded that average intake values in 67 (of 175 countries) exceed health protective guideline values.

Even when we have data on mercury levels in fish, the amounts can vary tremendously making it challenging to rely on database of average values. For example, a global assessment of 92 fish species from 26 countries reveals significant differences in mercury levels within a given species with key drivers being trophic position, size class of the fish, latitude, and ocean basin (Buck et al. 2019). A scan of available minimum and maximum mercury levels in the US FDA database reveals that the ratio varies from 2.2 (white croaker) to 200.8 (grouper) (US FDA 2022). Such findings raise concerns when using default mercury concentrations in risk assessment and management activities.

Consumption patterns are also not static. For example, the Food Balance Sheet (FAOSTAT) shows that increasing per capita consumption of fish and shellfish is a global trend, but especially in Asia and Oceania (U.S. FDA 2014), where fish-eating habits are naturally strong, there is a marked increase as living standards and economic access improve. It is interesting to note that in some of the populations in which mercury issues have been raised that consumption is declining. In Sects. 3.2 and 3.3, we detailed the dietary transition underway in the Faroe Islands and Arctic regions, respectively. In the Seychelles, there has also been a decline in fish consumption over the years. In 1995, pregnant women reported consuming 12 meals of fish per week and in 2015, there was an average of 8.5 meals of fish per week. Furthermore, data collected from the Seychelles Heart Study in 2004 showed that about 70% of study participants consumed fish daily, which decreased to around 25% of participants consuming fish daily in 2013 (Bovet et al. 2008).

#### Cultural considerations

Fish and food are cornerstones of all cultures and provides more than 4.5 billion people with about 15% of the average per capita intake of animal protein (Béné et al. 2015). As discussed in  "1975 to 2000: Toxicological studies on highly exposed populations" section, the contamination of culturally important food items (especially among Indigenous communities in the Arctic and Amazonian regions) raises broader social issues ranging from identity and spirituality to economic and recreational opportunities and food security.

In small-island states, seafood is a key item for nutrition as well as the economy. The Seychelles population consumes a wide variety of sea food found from reefs, plateau as well as fish found in the offshore. Thus, it is important to understand the safety of such high fish consumption and what are the micronutrients as well as risks of exposing to contaminants. A recent study, the SEYFISH project, was conducted to provide important baseline information on the micronutrient supply and contaminant exposure in edible parts of a total of 54 captured fisheries species from Seychelles coastal and offshore waters majority (Sabino et al. 2022). Seychelles marine resources have total mercury levels below the EU recommended limits, which is 1 μg/g for large pelagic and 0.05 μg/g for all other species. This level is considered low and comparable to commercially available fish in developing countries. Mercury was found to be lowest in benthic crustacean species. Importantly, the study provided valuable nutrition information on the accumulation of essential and non-essential trace elements in these different types of fish consumed by the Seychellois population.

Cultural connections and observations made in Japan, which has a rich history of whale harvesting, have similarly been made to pilot whales in the Faroe Islands as discussed earlier ("Faroe Islands" section). Residents living in the coastal town of Taiji, Japan, where traditional whaling began 300 years ago, continue to commercially hunt bottlenose dolphins (*Tursiops truncatus*), short-finned pilot whales (*Globicephala macrorhynchus*), Risso's dolphins (*Grampus griseus*) and striped dolphins (*Stenella coeruleoalba*). A previous study conducted in 2010 reported that the geometric mean of the hair mercury levels of 50 residents in Taiji was 15.0 μg/g and that their hair mercury levels correlated was correlated to frequent consumption of whale meat (Endo and Haraguchi 2010). The National Institute for Minamata Disease in Japan investigated the impact on whale food culture and clinical neurological symptoms (e.g., muscle weakness, rigidity, coordination, gait) in 194 residents who were accustomed to consuming whale meat (Nakamura et al. 2014). The geometric mean of hair mercury levels was 14.9 µg/g, which was 8.2 times higher than that of the Japanese population (Yasutake et al. 2003).

Across Africa, consumption of fish is widely practiced, and these are usually freshwater or marine fish that are consumed dried or fresh. The fish sector contributes to socioeconomic growth and helps alleviate poverty and improve the livelihoods of people in Africa (Chan et al. 2019). Given such fish consumption patterns, one would assume that methylmercury exposures are relatively high across the African continent. However, there are surprisingly low mercury levels in fish across Africa (Hanna et al. 2015). An examination of the few human biomonitoring studies tends to support this as the levels of mercury in blood or hair are also relatively low (Basu et al. 2018). One aspect that makes it difficult to ascertain fish consumption is that many African cultures do not eat fish as “discrete” food but mixed into soups by many thus making it difficult to gauge exposures. Another aspect that adds to the difficulty is that consumption patterns are not homogeneous across African regions as a result of accessibility, cultural preferences, and social preferences (Chan et al. 2019). More research is needed on mercury exposures across Africa.

#### Global change considerations

Global change (e.g., population growth, climate change) has led to large alterations in the availability of fish and seafood, which in turn will undoubtedly influence exposure of human populations to mercury. In 2002, approximately 76% of the world’s fisheries were used for human consumption, and at least 70% of global fish stocks are predicted to be either completely exploited, over exploited or recovering from depletion (FAO 2004; Brander 2007). Fishing poses a threat to global fish production and the impacts of fishing and climate change are interrelated (Brander 2007). For example, fishing causes a reduction in the age, size, and biodiversity of marine ecosystems making such environments more vulnerable to climate change. Modeling studies from the Gulf of Maine (USA) have estimated changes in mercury levels in Atlantic cod, spiny dogfish and Atlantic bluefin tuna to overfishing and temperature changes (Schartup et al. 2019). From the 2021 AMAP report, researchers investigated the importance of climate change on fish mercury levels and concluded that “it is clear that the effects of climate change on fish mercury concentrations are complex and difficult to predict” (Chételat et al. 2022).

### Artisanal and small-scale gold mining (ASGM)

The artisanal and small-scale gold mining (ASGM) sector is estimated to employ between 14 and 19 million people worldwide (Steckling et al. 2017). Upwards of 100 million people live in ASGM communities predominantly within East and Southeast Asia, Sub-Saharan Africa, and South America (WHO 2013; UNEP 2019a; Dórea 2021). In ASGM communities, the use of mercury to yield gold is of particular concern to human health (Gibb & O’Leary 2014; Steckling et al. 2017; UNEP 2019a). Once the gold-containing silt is ground into a powder form, elemental mercury is used to help form a stable amalgam (Esdaile & Chalker 2018). The amalgam is then subsequently burned to release the mercury to liberate the gold (Gibb and O’Leary 2014). During the amalgamation process, inorganic mercury is emitted, and the vapors may pose both an occupational and environmental threat to miners and area residents. In addition, mercury from mishandled mine tailings can contaminate soils and water. These tailings are often improperly maintained and eventually discharged into water systems or stored in mining pits without treatment (Rajaee et al. 2015). Mercury contamination within human populations and ecological components has been observed in many ASGM sites in countries including Brazil (Lebel et al. 1998), Indonesia (Bose-O’Reilly et al. 2010a, b), Tanzania (Bose-O’Reilly et al. 2010a, b), and Ghana (Basu et al. 2015a, b; Rajaee et al. 2015). In the Amazon rainforest, alluvial gold extraction occurs in riverbanks and in river sediments which involves heavy digging and diving activities, respectively (Dórea 2021).

In ASGM sites, biomonitoring studies have shown high exposures to mercury compounds (e.g., elemental mercury and methylmercury) among miners and area residents (Gibb and O’Leary 2014; Bose-O’Reilly et al. 2017). For example, the 2018 UN Global Mercury Assessment synthesized available data on mercury concentrations in urine samples from ASGM workers worldwide and found that the mean levels were approximately 30 to 200-fold higher than levels measured in general population (Basu et al. 2018). Mercury exposure within ASGM communities has been linked with neurotoxicity and other adverse health outcomes notably associated with elemental mercury poisoning (e.g., erethism, irritability, insomnia, severe salivation, gingivitis, tremors, kidney disease, acute gastrointestinal effects, and in case of direct effects, chemical pneumonitis and pulmonary edema) (Gibb & O’Leary 2014; Steckling et al. 2017).

ASGM workers worldwide tend to be relatively young males, though women and children are often found in many of these sites and nearby communities. The International Labour Organization (ILO) approximate that 4–5 million women and children participate in the ASGM sector across 70 countries worldwide (UNIDO 2007; Telmer and Veiga 2009). Although women make up a substantial portion of the ASGM sector, women often face gendered stereotypes as they are seen as incapable of performing certain tasks or manage resources (IGF/IISD, 2018). Additionally, women are also prohibited from the pit areas and thus unaware of the consequences of the minerals that they are mining (IGF/IISD, 2018). Child labor is also prevalent in many ASGM sites. Children are affected by mercury exposure in these communities as several studies have found associations between increased mercury levels and neurological abnormalities (e.g., increased deep tendon reflexes, poor leg coordination, decreased performance on visuospatial organization tests, and reduction in motor function, attention, visual contrast sensitivity, and manual dexterity). For example, a study conducted in the Philippines reported on associations between mercury levels and adverse neurological abnormalities within children residing near a gold mill and a processing plant (WHO 2013). Additional health concerns beyond mercury exposure exist among community members including dust and noise exposure, unsanitary working conditions, and lack of personal protective equipment (PPE) (Gibb and O’Leary 2014; Basu et al. 2015a, b). There is little known about mercury’s interaction with these and other stressors typically found in ASGM sites.

ASGM represents one of the most hazardous work environments worldwide and these work environments greatly differ from one another. Mercury exposure at ASGM sites can vary widely since sites are different in structure, size, and ecosystem characteristics. Some ASGM sites are rudimentary operations with no safety protocols, whereas others mimic industrial operations with state-of-the-art equipment and heavy machinery. While in some communities, exposures may be considered as para-occupational (i.e., processing takes place inside or near the home), and there are other situations in which the ASGM sites are far removed from communities. Concerning the latter, artisanal gold miners may sell their gold to shops in the city, which are typically located in the urban core. The gold is often processed further inside the shops to release any residual mercury from the amalgamation process. Gold shop workers have reported on mercury symptoms related to mercury intoxication including dizziness, tremors, and insomnia (Akagi et al. 1995). Urban centres of Segovia (Colombia) and Andacollo (Chile) with gold shops have measured mercury in the air at levels of 1.26 and 0.338 mg/m^3^, respectively (Cordy et al. 2013). According to WHO standards, these are relatively high mercury levels and thus area residents are at risk of adverse health impacts.

The Minamata Convention on Mercury aims to assess ASGM activities that are more than insignificant to develop and implement a national action plan (NAP) that includes public health approaches to protect vulnerable populations (i.e., Article 7 and Annex C). As mentioned by the Minamata Convention (Annex C 1-c), the International Labour Organization (ILO) and other researchers, formalizing the ASGM sector is important in addressing these issues within the ASGM sector (ILO 1999; Siegel and Veiga 2009; Basu et al. 2015a, b;). In many countries that participate in ASGM, approximately 70 to 80% of miners are informal in their practice (IGF/IISD, 2018). Although there are few empirical studies of this notion, the formalization of this sector will help improve health and safety ASGM sites. For example, a recent study from Ghana found that the median urinary mercury concentration among those from unregistered mines was nearly threefold higher than those from the registered mines (18.5 versus 6.6 mg/L) (Ovadje et al. 2021). A study found that the injury rate was higher between miners working in unlicensed mines in comparison to licensed miners in a mining region in Ghana (Calys-Tagoe et al. 2017). In addition to physical injuries, findings suggest that licensed mines' informal mining creates larger disparities in health and environmental issues, and ultimately that licensed mines have the potential to be safer for human and environmental health (IGF/IISD 2018).

### Skin-lightening products

Skin-lightening is the process of using chemical products to obtain a lighter skin complexion (Gillbro & Olsson 2011). The practice of skin-lightening is a global phenomenon that is widely practiced among women and men in parts of Asia, Africa, and the Caribbean (Mahé et al. 2007; Dadzie and Petit 2009; Uram et al. 2010; Lewis et al. 2011; Gbetoh and Amyot 2016). Many individuals in these communities believe that having lighter skin increases attractiveness, self-esteem, marriage prospects, and social and occupational mobility (Mahe et al. 2003; Ly et al. 2007; Hunter 2011; Thomas 2012; Dlova et al. 2015).

Historically, inorganic mercury salts (e.g., mercurous chloride (calomel), mercuric chloride, mercurous oxide, ammoniated mercuric chloride and mercuric iodide, ammoniated mercury) were added as an active ingredient to cosmetics and eventually into skin-lightening products (Al-Saleh and Al-Doush 1997; Risher and DeWoskin 1999; Risher and De Rosa 2007; Olumide et al. 2008; McKelvey et al. 2011; Copan et al. 2015). Inorganic mercury is readily absorbed into the skin and can interfere with the copper enzyme needed for tyrosinase activity, inactivating the melanocyte enzyme responsible for melanin production, preventing the skin from producing melanin (Denton et al. 1952; Lerner 1952; Sun et al. 2017; Chen et al. 2020). Implications of mercury-added skin-lightening cosmetics on human health are dependent on factors including dermal absorption rate, frequency and duration of usage, and mercury concentration in a given product (Park and Zheng 2012; Rice et al. 2014; Gbetoh and Amyot 2016). However, prolonged use of mercury-added skin-lightening cosmetics may lead to nephrotoxicity, contact dermatitis, and peripheral neuropathy (Palmer et al. 1998; Chan 2011; Ladizinski et al. 2011). Many women who use these products are of child-bearing age, and the potential transfer of mercury from mother to fetus could have implications resulting in neurological and nephrological disorders (Counter and Buchanan 2004; Bose-O’Reilly et al. 2010a, b).

Many governmental agencies worldwide have implemented regulations for usage of mercury in skin-lightening cosmetics. The Minamata Convention mandates that all Parties must prohibit the manufacture, import, and export of cosmetics including creams and soaps with a mercury concentration that exceeds 1 μg/g mercury by 2020 (UNEP 2019b). Despite the restrictions on mercury, skin-lightening products containing mercury enter countries through informal pathways (e.g., online sales) making it difficult for compliance with regulations (Glenn 2008; Bocca et al. 2014).

Quantitative data on exposure to inorganic mercury from skin-lightening cosmetics are limited. Though a recent assessment using a systematic review methodology, Bastiansz et al. (2022) compiled and analyzed evidence from 41 peer-reviewed scientific papers from 22 countries worldwide published between 2000 and 2020. In total, mercury concentration values from 787 skin-lightening product samples (overall pooled central median mercury level was 0.49 μg/g, IQR: 0.02–5.9) and 1,042 human biomarker measurements from 863 individuals were obtained. This research also synthesized usage information from 3,898 individuals, and self-reported health impacts associated with using mercury-added products from 832 individuals. It is evident that mercury still widely exists as an active ingredient in skin-lightening products and that there is large variability in human exposures.

### Dental amalgam

There remains concern worldwide among some groups about the potential health effects of exposure to mercury vapor that may be released from dental amalgam restorations. This form of restoration has been used for over 100 years, and most standard formulations contain approximately 50% elemental mercury. Expert panels from across Europe, the United States, Canada, and Australia among others have concluded that there is no strong scientific evidence to make a causal link between dental amalgam restorations and adverse health outcomes except for some rare cases of hypersensitivity in certain individuals (Brownawell et al. 2005). Nonetheless, research continues in this area. Foremost is that mercury exposures have been steadily declining among dental professionals in many regions. For example, in Japan, the about 5 200 kg of mercury was used in amalgams in 1970, and this has reduced to ~ 700 kg in 1999, ~ 100 kg in 2006, and ~ 20 kg in 2010 (Japan dental chamber association 2010). Through a biomonitoring program run by the American Dental Association on their membership, researchers have shown a decrease of nearly tenfold in the urinary mercury values between 1975 and 2012 (Goodrich et al. 2016). Dental amalgam constitutes an important source of exposure to elemental mercury in many population groups, with estimates of daily intake from amalgam restorations ranging from 1 to 27 μg/day with the majority of dental amalgam holders being exposed to less than 5 μg mercury/day (WHO 2003). Dental amalgam remains the prevailing dental restoration material in most countries worldwide. However, the situation differs between countries and regions. For example, in reviewing data from the Minamata Initial Assessments (MIAs), only encapsulated forms were reported being used across 35 European countries (with the majority of Europeans using mercury free fillings), whereas in data from 14 African nations, there was a combination of both liquid mercury and encapsulated forms being used (UNEP 2018). From these MIAs, the amount of mercury reportedly used also varies significantly with estimates ranging from 25 to 56 t/y in the EU to 10 kg to 5t in Africa.

### Contaminated sites

Areas adjacent to anthropogenic activities that either use mercury and/or release it as a by-product may be contaminated and thus pose health risks to workers and community members. Major source sectors, based on data compiled for the 2018 UN Global Mercury Assessment (UNEP 2019a), include ASGM (see "Faroe Islands" section; 37.7% of emissions from anthropogenic source sectors), coal combustion power plants (13.1%), cement production (10.5%), and non-ferrous metal production (mainly zinc, copper, and lead; 10.3%). In addition, mercury contamination risks at chlor-alkali plants, pulp and paper mills, battery and light manufacturing centers, and mercury mines have been well documented (Risher and DeWoskin 1999; Jarosińska et al. 2008). In terms of the location of these sites, most are in Europe (40%) and Asia–Pacific (38%) (UNEP 2019a). Compared to other population groups, there are fewer human health projects concerning groups that either work and/or reside near contaminated sites. Community members of anthropogenically mercury-polluted areas can be constantly exposed to mercury vapor and inorganic mercury and also to methylmercury from consumption of local fish and locally grown foods that have been found to contain increased mercury concentrations (Miklavčič et al. 2013; Jiménez-Oyola et al. 2020). Mercury concentrations in human biomarkers in such areas are shown to be high in comparison with controls and reference values. Due to exposure to mixture of mercury compounds at mercury-contaminated sites, the use of appropriate exposure biomarkers and adequate interpretation of results are crucial. It is important to consider that people in such areas are exposed not only to mercury, but also to noise, dust, cadmium, arsenic, lead, etc., that also contribute to adverse health effects and require further investigation (Ha et al. 2016; Branco et al. 2017). From the 2018 UN Global Mercury Assessment, mercury levels among populations from contaminated sites were approximately 3 and 1.5 times higher for urine and blood, respectively, when compared to background populations (Basu et al. 2018).

### Electronic waste recycling

Electronic waste (e-waste) results from the disposal of electrical equipment and electronic waste (EEE) (i.e., products with circuits or electrical components and a power or batter supply) (Heacock et al. 2018). The rapid production and use of electrical products, with relatively few repair and recycling options, has increased the generation of e-waste growing by approximately 3–5% annually, with Asia generating the largest amount of e-waste overall (24.9 Mt total and 5.6 kg per capita) and Europe generating the largest amount of e-waste per capita (12 Mt total and 16.2 kg per capita) (Forti et al. 2020). In low- and middle-income countries, approximately 80% of global e-waste is informally handled through “backyard recycling” that lacks adequate management and safety infrastructure, creating unsound handling of heavy metals including mercury (Forti et al. 2020).

Several EEE products contain mercury (e.g., fluorescent lights, switches, batteries, phones, and computers) and after these products are burned and broken down for recycling, mercury may be released into the surrounding environment (Kyere et al. 2017; Forti et al. 2020). Through a systematic review of the scientific literature, the average mercury concentration in 1,103 e-waste products was 0.65 μg/g, with the highest values found in fluorescent lights, batteries, and LCD backlights (Aubrac et al. 2022). In this same study, which examined 78 papers published between 2005 and 2022, mercury concentrations were reported from diverse media including water, dust, food, air, and plants. The work also synthesized mean mercury levels in human biomarker samples (i.e., 0.60 μg/L from 399 blood samples, 0.7 μg/L from 128 serum samples, 0.104 μg/L from 273 urine samples, and 0.72 μg/g from 151 hair samples). These mercury levels are higher than those found in dental workers but less than those working in ASGM. Similar to ASGM, there is great diversity in the structure, size, and operation of e-waste recycling sites and thus a need for more studies in this area to appreciate the full range of mercury exposure and risk scenarios.

## Concluding remarks

In this final section of the paper, we conclude with proposed next steps to conduct mercury health research in the post-Minamata Convention era (Table 1). We focus first on what we—as health researchers—can contribute to the Minamata Convention. Second, we focus on the need to continue to study the health risks of mercury pollution given that investigations that enable us to both deepen and broaden our understandings can have broader impacts in the field and beyond.

### Evaluating the effectiveness of the Minamata Convention

Article 1 of the Minamata Convention represents a global commitment by governments to reduce the use and environmental release of mercury in order to protect human health and the environment. Health features prominently in other Articles of the Convention including Article 16 titled “Health Aspects,” and Article 19 titled “Research, Development and Monitoring” which emphasizes a need to focus on vulnerable populations. It is important for Parties to educate and make the public aware of environmental and human health effects of mercury and mercury compounds, as well as alternatives or management solutions to phase-out mercury.

To protect human health means to ensure that exposures to mercury are reduced to levels that pose insignificant threats. As such, gauging exposures to mercury represent the most objective, cost-effective, and sensible approach to evaluating the effectiveness of the Convention (vs. risk or hazard-based approaches). Human biomonitoring of mercury (i.e., mercury measures in hair, urine, and/or blood) is well understood, practiced by some national governments, and can help assess the efficacy of policy actions (WHO 2018; HBM4EU 2019; UNEP 2019a), and as such has been proposed as a key measure in evaluating the effectiveness of the Minamata Convention. Human biomonitoring data can help to address core policy questions that drive effectiveness evaluation. First, quality measures of mercury levels in human biological samples provide direct evidence of exposure in a given population from which judgements can be made. Second, such measures may offer insights into possible sources and routes of exposure from which attributions may be deduced. Third, temporal changes can be gleaned if monitoring is repeated in the same population over time. Fourth, biomonitoring data can be inputted into established risk assessment frameworks to estimate health impacts including burden of disease, as well as to assess the efficacy of different risk management strategies.

**Fig. 1:**
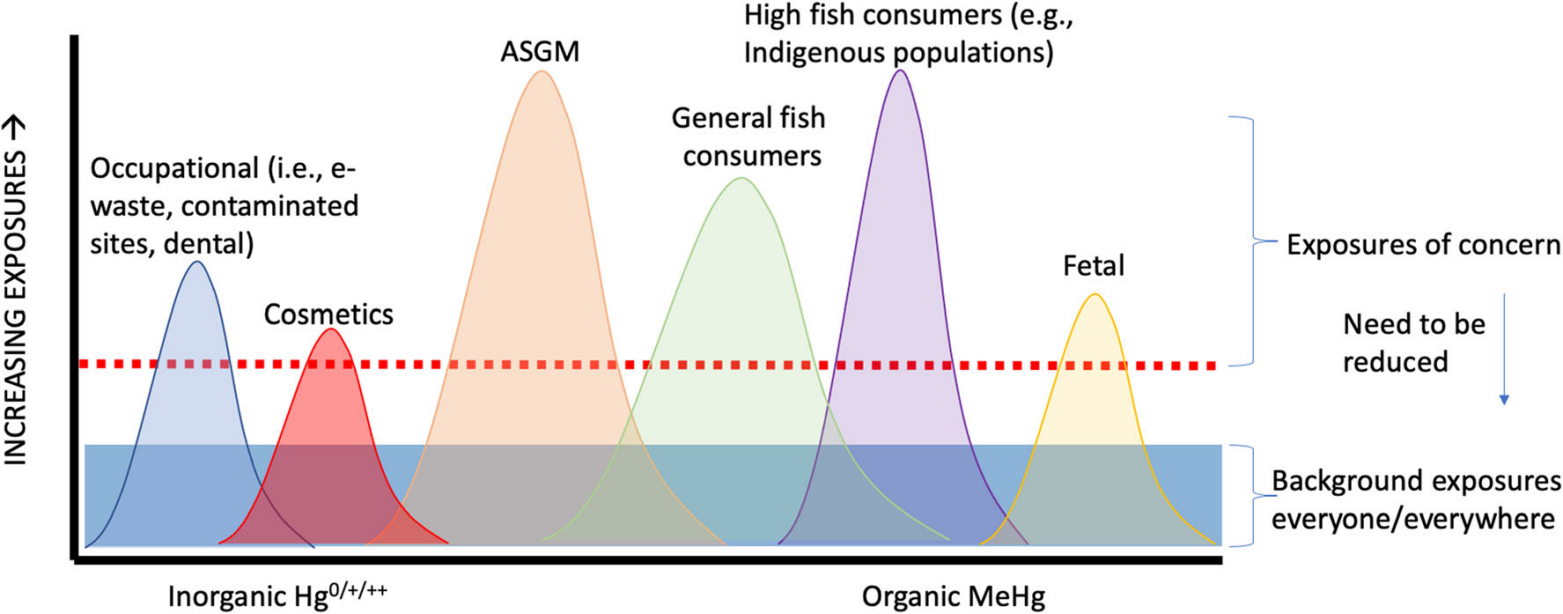
Population groups with relatively high exposures to mercury and its compounds

In terms of identifying populations that may be at risk of mercury exposure and health impacts, a guidance document was produced through a collaboration between the UN Environment Programme and the WHO (UNEP and WHO 2008). From the 2018 UN Global Mercury Assessment report (Basu et al. 2018), four populations of concern were identified based on existing datasets: (1) Arctic populations (mainly Inuit) who consume fish and marine mammals; (2) tropical riverine communities (especially Amazonian) who consume fish, and in some cases may be exposed to mining operations; (3) coastal and/or small-island communities (including Indigenous Peoples) who rely substantially on seafood; and (4) individuals who either work or reside among ASGM sites. Such populations were discussed in the current paper, though there is a need to broaden investigations to other population groups ranging from members of the general public who experience chronic exposures to background levels to relatively under-studied groups with elevated exposures (e.g., users of skin-lightening products, populations associated with contaminated sites, e-waste recyclers) (Fig. 1).

National programs to understand mercury exposures and risk are particularly important as they may yield robust data (i.e., large sample size, use of quality laboratory measures) that is representative of a particular place. There have been plenty of studies on methylmercury exposures across countries within North America, for example. Using data from the 2012 National Health and Nutrition Survey of Mexico involving 10 096 participants, the mean annual intake of seafood was 3.65 kg with canned tuna, sunfish, shrimp being the top 3 food items. Canned tuna and school shark contributed to 75% of the population’s exposure to methylmercury (Cantoral et al. 2017). In Canada, using data from the Canadian Community Health Survey, the average Canadian consumed 6.2 kg of fish per year with salmon, tuna, and shrimp being most common (Hu and Chan 2021). National dietary surveys can help identify species that contribute to population-level exposures and thus may be helpful for targeted interventions. Some of these national programs also conduct human biomonitoring. For example, an analysis of blood mercury levels from the Canadian Health Measures Survey (CHMS, Canada) and US National Health and Nutrition Examination Survey reveals annual decreases of ~ 2% over the past 10 years.

An additional aspect to consider when assessing the effectiveness of measures developed to reduce mercury exposure should be based on a coherent framework for communicating fish consumption advice to vulnerable groups, especially pregnant women. For example, within the framework of the European project "HBM4EU-MOM" ("Control of methylmercury in expectant mothers with the help of appropriate nutritional advice for pregnancy"), an intervention study was carried out for the first time, which developed a coordinated framework for communicating advice on fish consumption to pregnant women. This study also demonstrated the usefulness of mercury biomonitoring for the evaluation of the effectiveness of fish consumption advice (i.e., to promote fish consumption in a way that maintains consumers below the health-based guidance values of EFSA and the WHO) (Katsonouri 2022).

### A need for more research

With a global policy instrument now in place one may instinctively conclude that additional health research on mercury pollution is unwarranted. Experts in the field, including us, challenge this notion and propose that now is the time to further escalate our research efforts so as to realize a wider range of benefits and broader understandings (Evers et al. 2016; Gustin et al. 2016; Basu 2018; Chen et al. 2018).

First, we have amassed a large knowledge base for mercury, and from this, we have been able to break new frontiers not only in mercury sciences but also across the health sciences (Eagles-Smith et al. 2018). For example, in recent years, researchers have documented that: (a) mercury’s seven stable isotopes, when measured with highly precise and accurate methods and coupled with clever calculations, can reveal new insights into source, fate, bioaccumulation pathways to better understand human exposures (Li et al. 2022); (b) the toxic impacts of mercury extend far beyond the nervous system with evidence of cardiovascular and immune system impacts (Ha et al. 2016); (c) gene-environment interaction studies, such as genetic polymorphisms or epigenetics, may help explain inter-individual differences in exposure and risk, as well as deepen understanding of toxic mechanisms of action and life stage differences (Basu et al. 2014; Tratnik et al. 2017); (d) socioeconomic costs of mercury pollution are in the billions of dollars per year (Trasande et al. 2005), from which similar cost impacts of other chemicals have been performed (Landrigan et al. 2018). These examples, and many more, arising from mercury health studies have helped increase the understanding of core principles that underpin diverse scientific fields such as biogeochemistry, genetics, human behavior, and economics. These observations have also been extended to help increase the understanding of risks posed by other contaminants.

Second, mercury continues to complicate matters of public health (e.g., seafood consumption, amalgam) and economic prosperity (e.g., mining), and we remain challenged in our ability to understand, manage, and explain the risks and benefits. We are just starting to scratch the surface of the overall burden of disease caused by mercury with some examples emerging from ASGM (Steckling et al. 2017) and prenatal exposures (Grandjean and Bellanger 2017). Mercury exposure occurs in combination with other toxic substances as well as non-chemical stressors (e.g., nutrition) and factors (e.g., genetics), and the cumulative effects are poorly resolved. Even the health implications of combined exposure to both organic and inorganic mercury (i.e., mercury-mercury mixtures) are not well known. As discussed earlier, the neurodevelopmental impacts of mercury exposure have been documented in some cases, though further research is needed to see how early-life exposures extend into potential effects across the entire lifespan. Prenatally exposed members of some cohort studies (e.g., Seychelles and Faroe Islands) are entering adulthood, from which necessary studies continue to unfold. All of these aspects are important to consider when performing risk–benefit analyses.

Finally, despite thousands of scientific articles and decades of research, there are relatively poorly understood yet important aspects concerning mercury’s human health risk that remain unresolved. Notably there are very few studies from low- and middle-income countries even though population groups from these areas remain among the most vulnerable worldwide. In affluent countries, most studies tend to focus on methylmercury exposure from fish/seafood consumption, in contrast to low- and middle-income countries where exposures tend to be more diverse (Dórea 2021). A central tenet of this paper is that source-exposure scenarios differ worldwide as do pertinent socio-environmental variables and population characteristics. Though we tend to draw analogies and infer external validity through comparison results of exposure-outcome studies ranging from Inuit adults in the Arctic to prenatally exposed newborns in a small-island nation to ASGM workers in Latin America to members of the general population in Asian cities, there are stark differences that must be considered and thus a need to focus on the generation and use of context-specific data.

## Data Availability

Not applicable.

## Footnotes

1 https://www.youtube.com/watch?v=Ys94Q5Xvww8.
